# Repurposing FDA-approved drugs for anti-aging therapies

**DOI:** 10.1007/s10522-016-9660-x

**Published:** 2016-08-02

**Authors:** Terry W. Snell, Rachel K. Johnston, Bharath Srinivasan, Hongyi Zhou, Mu Gao, Jeffrey Skolnick

**Affiliations:** School of Biology, Georgia Institute of Technology, Atlanta, GA 30332-0230 USA

**Keywords:** Aging, Re-purposing drugs, Rotifera, Lifespan, Healthspan, TRP genes

## Abstract

There is great interest in drugs that are capable of modulating multiple aging pathways, thereby delaying the onset and progression of aging. Effective strategies for drug development include the repurposing of existing drugs already approved by the FDA for human therapy. FDA approved drugs have known mechanisms of action and have been thoroughly screened for safety. Although there has been extensive scientific activity in repurposing drugs for disease therapy, there has been little testing of these drugs for their effects on aging. The pool of FDA approved drugs therefore represents a large reservoir of drug candidates with substantial potential for anti-aging therapy. In this paper we employ FINDSITE^comb^, a powerful ligand homology modeling program, to identify binding partners for proteins produced by temperature sensing genes that have been implicated in aging. This list of drugs with potential to modulate aging rates was then tested experimentally for lifespan and healthspan extension using a small invertebrate model. Three protein targets of the rotifer *Brachionus manjavacas* corresponding to products of the transient receptor potential gene 7, ribosomal protein S6 polypeptide 2 gene, or forkhead box C gene, were screened against a compound library consisting of DrugBank drugs including 1347 FDA approved, non-nutraceutical molecules. Twenty nine drugs ranked in the top 1 % for binding to each target were subsequently included in our experimental analysis. Continuous exposure of rotifers to 1 µM naproxen significantly extended rotifer mean lifespan by 14 %. We used three endpoints to estimate rotifer health: swimming speed (mobility proxy), reproduction (overall vitality), and mitochondria activity (cellular senescence proxy). The natural decline in swimming speed with aging was more gradual when rotifers were exposed to three drugs, so that on day 6, mean swimming speed of females was 1.19 mm/s for naproxen (P = 0.038), 1.20 for fludarabine (P = 0.040), 1.35 for hydralazine (P = 0.038), as compared to 0.88 mm/s in the control. The average reproduction of control females in the second half of their reproductive lifespan was 1.08 per day. In contrast, females treated with 1 µM naproxen produced 1.4 offspring per day (P = 0.027) and females treated with 10 µM fludarabine or 1 µM hydralazine produced 1.72 (P = <0.001) and 1.66 (P = 0.001) offspring per day, respectively. Mitochondrial activity naturally declines with rotifer aging, but *B. manjavacas* treated with 1 µM hydralazine or 10 µM fludarabine retained 49 % (P = 0.038) and 89 % (P = 0.002) greater mitochondria activity, respectively, than untreated controls. Our results demonstrate that coupling computation to experimentation can quickly identify new drug candidates with anti-aging potential. Screening drugs for anti-aging effects using a rotifer bioassay is a powerful first step in identifying compounds worthy of follow-up in vertebrate models. Even if lifespan extension is not observed, certain drugs could improve healthspan, slowing age-dependent losses in mobility and vitality.

## Introduction

There is great interest in finding drugs capable of extending human lifespan and healthspan (Armanios et al. 2015). Compounds are sought that are capable of modulating multiple aging pathways, thereby preventing a broad-spectrum of age-related diseases. Conserved aging pathways have been identified using experimental animal models (de Cabo et al. 2014), and some compounds capable of delaying the onset and progression of aging have been identified; these include spermidine, rapamycin, metformin and resveratrol.

One of the most effective strategies for drug development is the repurposing of existing drugs that have been approved by the FDA for human therapy (Ashburn and Thor 2004; Pantziarka et al. 2014). FDA approved drugs have known mechanisms of action and have been thoroughly screened for safety. Moreover, many drugs have much broader ranges of action than their licenses suggest. FDA approved drugs are likely to have low risks, so that even small benefits might provide an acceptable risk/benefit ratio. Although there has been extensive commercial activity in repurposing drugs for disease therapies (Naylor and Schonfeld 2015), there has been little testing of these drugs for their effects on aging. The pool of FDA approved drugs therefore represents a large reservoir of drug candidates with substantial potential for anti-aging therapy. Repurposing existing drugs could have a rapid impact on human aging if drugs can be shown to have efficacy in slowing the progression of age-related diseases. A strategy for rapidly finding such drugs is to screen the suite of existing drugs computationally and then to test the predictions experimentally using a short-lived invertebrate model. Promising results can then be followed up in vertebrate and mammalian experimental models, then confirmed in human clinical trials.

 Rotifers are experimentally tractable animal models for studying the biology of aging and representatives of the supra-phylum Lophotrochozoa (Snell 2014). The brachionid rotifers that we study are about 0.4 mm long aquatic herbivores, capable of both asexual and sexual reproduction. Their lifespan in the laboratory at 22 °C is about 2 weeks, enabling rapid screening of many treatments using cohort life tables (Snell et al. 2014). For example, libraries of natural products from marine algae have been screened for bioactivity using this rotifer model (Snare et al. 2013). Rotifers, therefore, are useful animal models for initial drug screening for life and healthspan extension before moving to vertebrate models.

Several rotifer genes and pathways capable of extending lifespan have been identified (Oo et al. 2010; Ozaki et al. 2010; Snell et al. 2012, 2014b; Gribble and Welch 2012; Snell and Johnston 2014; Johnston and Snell 2016). Prominent among them are nutrient sensing and stress resistance pathways. A less appreciated pathway with substantial effects on aging is the temperature-sensing pathway. Like many animals, rotifer lifespan is extended by exposure to moderately lower temperatures as a result of combined thermodynamic effects on metabolic reactions and changes in gene expression (Johnston and Snell 2016). These authors used a combination of life table experiments, stressor challenge experiments, and RNAi knockdown treatments to identify putative temperature sensing genes likely involved in regulating aging rates. Four of 12 temperature-sensing genes were identified as especially promising drug targets for life and healthspan extension. Among these, we have explored three rotifer temperature sensing genes and computationally investigated drug binding partners with the potential to modulate their expression.

Many bioinformatics and computational approaches for drug-protein interaction discovery have been developed (Cavasotto and Orry 2007; Cheng et al. 2012; Gottlieb et al. 2011; Laarhoven and Marchioro 2013; Lee and Zhang 2011; von Eichborn et al. 2011; Yamanishi et al. 2008; Yamanishi et al. 2010). Some methods use protein sequences and a priori knowledge of binding drugs of a protein of interest (Laarhoven and Marchioro 2013; Yamanishi et al. 2010), whereas docking-based approaches require high-resolution protein structures that in most cases are not available (Cavasotto and Orry 2007). The recently developed FINDSITE^comb^, a ligand homology modeling approach that has been extensively experimentally validated (Srinivasan et al. 2014; Zhou and Skolnick 2013), has several advantages over other state-of-the-art methods for predicting drug–protein interactions: First, it does not require known drug interactions for a protein target as is required by many sequence-based methods; second, it does not require high resolution protein structures required by docking-based methods; third, it is much more efficient than docking-based methods; and last, and most importantly, it has better accuracy for ranking drug–target interactions than other methods (Zhou and Skolnick 2013). FINDSITE^comb^ has been applied in the creation of the DR.PRODIS database (Zhou et al. 2015), which contains comprehensive drug–protein interactions predicted for the entire Human proteome.

The hypothesis of this paper is that FINDSITE^comb^ can identify drug candidates capable of life or healthspan extension, and that these can be rapidly tested experimentally using rotifers as an animal model.

## Materials and methods

### FINDSITE^comb^ algorithm for computational screening of FDA approved drugs

FINDSITE^comb^ (Zhou and Skolnick 2013) is a computational method that is capable of screening millions of compounds on a desktop computer within a few hours. The basic assumption of FINDSITE^comb^ is that similar pockets bind to similar ligands regardless of evolutionary relationship. However, evolutionarily related proteins have a high chance of being similar structurally. For a given protein of unknown ligands and unknown structure, FINDSITE^comb^ firstly builds a structure model of the protein target using state-of-the-art threading method (Skolnick et al. 2004; Zhou and Zhou 2005) and one of the best protein structure modeling approaches, TASSER (Zhang and Skolnick 2004). The modeled structure is subsequently employed in searching against a library of pockets with experimentally determined ligands [PDB holo structures (Bernstein et al. 1977)] in the FINDITE^filt^ component (Zhou and Skolnick 2013)—an updated version of FINDSITE approach (Brylinski and Skolnick 2008); and is then searched against a library of modeled structures of proteins having experimentally determined binders but no experimentally determined structure of the ligand–protein complex structures [DrugBank (Wishart et al. 2006) & ChEMBL (Gaulton et al. 2012)] in the FINDSITE^X^ (Zhou and Skolnick 2012) component. The ligands of the top 100 ranked pockets from PDB, of top ranked proteins from DrugBank & ChEMBL (called “template ligands”) are used as “seeds” to search against a compound library by a similarity-based approach. The ligands in the compound library are independently ranked by the three components (FINDSITE^filt^ using the PDB, FINDSITE^X^ using DrugBank & FINDSITE^X^ using ChEMBL) according to their similarity to the respective template ligands. The combined ranking gives a final prediction. In practice, the top 1 % of ranked ligands of the compound library are considered for further experimental test.

 In this work, three protein targets of *Brachionus manjavacas* corresponding to products of the transient receptor potential gene 7 (GARS01012197.1, TRP7), ribosomal protein S6 polypeptide 2 gene (GARS01003002.1, S6P), or forkhead box C gene (GARS01006072.1, FhBC), were screened against a compound library consisting of DrugBank drugs including 1347 FDA approved, non-nutraceutical molecules. Together with the ZINC8 (Irwin and Shoichet 2005) background, a total of 74,378 molecules are screened by FINDSITE^comb^. FDA approved drugs ranked within the top 1 % (i.e. higher than 740th) for each target are subsequently considered for further experimental validation. Candidate drugs are listed in Table 1.

**Table 1:** Drugs predicted to bind to proteins produced by three temperature sensing *Brachionus manjavacas* genes, TRP7, S6P, and FhBC

Gene	Drug	Ranking	Mechanism	Names	Source
TRP7	Tadalafil	12	PDE-5 inhibitor	Cialis, Adcirca	Selleck Chemicals
	Sildenafil	14	cGMP PDE-5 inhibitor	Viagra	Selleck Chemicals
	Vardenafil	15	PDE-5/PDE-1 inhibitor	Levitra	Selleck Vhemicals
	Enprofylline	16	Xanthine derivative, A1/A2 adenosine receptor antagonist		LGM Pharma
	Enoximone	20	PDE-3 inhibitor	Perfan	Sigma-Aldrich
	Amrinone	44	PDE-3 inhibitor	Inocor	Sigma-Aldrich
	Hydralazine	46	Smooth muscle relaxant- K-channel activator	Apresoline	Selleck Chemicals
	Lenalidomide	47	TNF-a secretion inhibitor	Revlimid	Selleck Chemicals
	Dyphylline	56	Xanthine derivative, adenosine receptor antagonist, PDE inhibitor	Dilor, Lufyllin	Selleck Chemicals
	Pentoxifylline	68	Xanthine derivative, adenosine receptor antagonist, PDE inhibitor	Trental	Selleck Chemicals
S6P	Lactulose	68	Synthetic disaccharide- promote gut bacterial growth		Selleck Chemicals
	Fludarabine	185	STAT1 activation inhibitor, DNA synthesis inhibitor	Fludara	Selleck Chemicals
	Naproxen	189	COX-1/COX-1 inhibitor	Aleve, Naprosyn	Selleck Chemicals
	Calcium gluceptate	236	Calcium supplement		Selleck Chemicals
	Fusidic acid	351	Bacteriostatic antibiotic–protein synthesis inhibitor		Sigma-Aldrich
	Adenosine	361	Purine nucleoside- energy transfer, signal transduction		Selleck Chemicals
	Vidarabine	362	Inhibits viral DNA synthesis	Vira-A	Selleck Chemicals
	Ibuprofen	381	COX-1/COX-1 Inhibitor	Advil, Dolgesic	Selleck Chemicals
	Mupirocin	408	isoleucyl tRNA synthetase inhibitor	Bactroban, Centany	Selleck Chemicals
	γ-Hydroxybutyric acid	444	CNS depressant- GHB agonist, weak GABA antagonist	Xyrem	Controlled Subst
FhBC	Lamotrigine	13	5-HT inhibitor, Na-channel blocker	Lamictal	Selleck Chemicals
	Mexiletine	21	Na-channel inhibitor	Mexitil	Selleck Chemicals
	l-Carnitine	29	Fatty acid transport, lipid breakdown		Selleck Chemicals
	Leucovorin	85	Rescues low levels of folic acid		Selleck Chemicals
	Fludarabine	119	STAT1 activation inhibitor, DNA synthesis inhibitor	Fludara	Selleck Chemicals
	Methotrexate	178	Folic acid metabolism inhibitor	Trexall	Selleck Chemicals
	Adenosine	218	Purine nucleoside- energy transfer, signal transduction		Selleck Chemicals
	Vidarabine	219	Inhibits viral DNA synthesis	Vira-A	Selleck Chemicals
	Nafarelin	293	GRH analogue, LH-RH agonist, decreases LH and FSH	Synarel	Sigma-Aldrich
	Colistin	320	Polymyxin antibiotic, breaks down bacterial membranes	Xylistin, Koolistin	Selleck Chemicals

### Rotifer culture

 All experiments were performed using the rotifer species *B. manjavacas*. This strain was originally collected from the Azov Sea in Russia, and has been continuously cultivated in the Snell laboratory since 1983, with resting eggs being periodically collected, dried, and stored. Before each experiment, *B. majavacas* resting eggs were hatched in 25 mL, 15 ppt artificial sea water (ASW, Instant Ocean) under constant fluorescent illumination (2000 lux) at 25 °C. Under these conditions, hatching begins after 18–20 h, resulting in a uniform cohort of neonates. Hatchlings were fed the green alga *Tetraselmis suecica* cultured in modified F medium (Guillard 1983) in a 560 mL chemostat with 25 % daily medium replacement, at 25 °C and constant fluorescent illumination of 2000 lux. All cultures were kept in percival I-41VL incubators to maintain stable environmental conditions (Table 2).

**Table 2:** Effect of TRP agonists and antagonists on *B. manjavacas* reproduction and lifespan

Compound	Target	Source	Conc. (μM)	Reproduction	Lifespan
Capsaicin	TRPV1 agonist, hot taste of chili peppers	Sigma-Aldrich	1	NS	NS
			10	NS	NS
Resiniferatoxin	TRPV1 agonist, analog of capsaicin	Sigma-Aldrich	0.5	NS	↓40 %
			1	NS	↓56 %
Icilin	TRPM8 Super-agonist, similar effects to menthol	Sigma-Aldrich	1	↑16 %	NS
			5	NS	NS
			10	NS	-
WS-12	Selective TRPM8 agonist	Sigma-Aldrich	1	↑6 %	NS
			5	NS	NS
Allyl isothiocyanate	TRPA1 and TRPV1 agonist, pungent mustard taste	Sigma-Aldrich	1–22 °C	NS	NS
			1–16 °C	–	↓12 %
HC030031	Selective TRPA1 blocker, lC_50_ = 5 μM	Sigma-Aldrich	5–22 °C	NS	NS
			5–16 °C	–	↓16 %
SB705498	Antagonizes heat activation of hTRPV1	Selleck	0.5	NS	NS
			1	NS	NS
AMG-517	Selective TRPV1 antagonist	Selleck	0.5	NS	NS
			1	NS	NS

### Experimental design and treatments

Full cohort life table experiments were conducted with 120 newly hatched female *B. manjavacas* per treatment. Animals were kept in 24-well plates, with each well containing five females in 1 mL of medium. The medium contained 6 × 10^5^ *T. suecica* cells/mL in 15 ppt ASW, any drug treatments, and 20 µM 5-fluoro-2-deoxyuridine (FDU), added to prevent the hatching of asexual eggs (Snell et al. 2014b). In experiments where drugs were dissolved in DMSO, the control medium also contained DMSO at the same concentration. Plates were incubated at 22 °C in the dark. Animals were checked daily and mortality was recorded until all animals were dead. All animals were transferred to new plates with fresh medium on day 8 to replenish their food supply, FDU, and drug treatment.

Reproductive life table experiments were performed in a similar fashion, with a few modifications. Single females were kept in each well of 24-well plates in 1 mL of medium, for a total of 24 females per treatment. The medium contained 2 × 10^5^ *T. suecica* cells/mL in 15 ppt ASW and drug treatment. No FDU was added to allow for normal egg hatching. Offspring were produced parthenogenetically and were counted and removed daily. The original maternal females were transferred to new plates with fresh medium on day 6. For reproductive screens exploring the effects of TRP effector drugs, offspring were counted and removed for only the first 3 days. The total number of offspring over 3 days was used to calculate the intrinsic population growth rate (r) for each treatment.

### Survival screens

Survival screens were conducted with a cohort of 84 *B. manjavacas* hatchlings per treatment and 164 hatchlings in the control. Animals were kept in 24-well plates with each well containing 7 females in 1 mL of medium. The medium contained 6 × 10^5^ *T. suecica* cells/mL in 15 ppt ASW, any drug treatments, and 20 µM FDU. Plates were incubated at 28 °C in the dark. On days 2, 4, and 6, 5 μL additional FDU (1 mg/mL) was added to each well to prevent the hatching of eggs. On day 6, 100 μL 6.6 × 10^6^ cells/mL *T. suecica* was added to each well to replenish food supply. On day 8 or 10, the number of living animals was counted and survival was recorded as average percent surviving in each well.

### Estimation of rotifer swimming speed

To analyze swimming speed at different ages, rotifer hatchlings were cultured in 6-well plates with approximately 40 females per well in 5 mL medium. The medium contained 6 × 10^5^ *T. suecica* cells/mL in 15 ppt ASW, any drug treatments, and 20 µM FDU. Plates were incubated at 22 °C in the dark. Animals were transferred to new plates with fresh medium on day 8. On days 0, 2, 4, 6, 8, 10, and 12, 15 females from each treatment were chosen randomly and transferred to 5 mL 15 ppt ASW to rinse away algae cells. These animals were then transferred to painted microscope slides, with five females in each painted well in 12 μL ASW. Swimming behavior in each well was recorded for 30 s at 10× magnification using a PixeLink camera attached to a stereomicroscope. The Tracker Video Analysis and Modeling Tool program (http://physlets.org/tracker/) was used to track the movement of each individual animal and calculate velocity in mm/s. Swimming speed was estimated for 6–10 individuals from each treatment at each time point.

### Estimation of mitochondrial activity

MitoTracker^®^ Red (Invitrogen), which has an excitation of 581 nm and an emission of 644 nm (Snell et al. 2014b), was used to measure mitochondrial activity. Female rotifers were cultured in 24-well plates with 6 × 10^5^ *T. suecica* cells/mL in 15 ppt ASW, appropriate drug treatments, and 20 µM FDU for 4 days. Plates were incubated at 22 °C in the dark. On day 4, animals were rinsed in 15 ppt ASW for 2 h to clear their guts and eliminate auto-fluorescent algae. After clearing, the animals were incubated in 5 μM MitoTracker^®^ Red in the dark for 30 min. The stain was then rinsed away with ASW. The rotifers were anesthetized with 1 mL club soda, fixed with 20 μL 20 % formalin, and rinsed with ASW. Images were taken at 200× magnification with an Alexa 568 nm excitation filter using a Zeiss Imager Z1 microscope with a 5 ms exposure. The average pixel intensity of each animal was measured using ImageJ. The entire animal was selected and measured, and the pixel intensity of the background was subtracted. Twenty animals were measured for each treatment.

### Statistics

Survival curves from full life tables were evaluated using the JMP Pro 11 (SAS Institute) reliability and survival analysis with a Wilcoxon’s test to compare control and treatments. Eight day survival screens were compared by ANOVA with Dunnett’s test to compare treatments to control. A similar analysis was used for swimming speed, MitoTracker, and reproduction.

## Results

The algorithm FINDSITE^comb^ (Zhou and Skolnick 2013) was used to generate a list of FDA approved drugs that are predicted to bind to proteins produced by the S6Ka, TRP7, and FhBC rotifer putative temperature sensing genes. Only the top 1 % of the strongest binders were considered. In practice this translates to ten drugs for each gene (Table 1). We selected four drugs to test for each gene, based on availability, price, and mode of action. Drugs like fludarabine that appeared on the list of two genes were favored. We first tested the effects of selected drugs from this list on rotifer asexual reproduction using a 3 day dose–response screen (data not shown). This allowed us to estimate the toxicity of each drug and the likely therapeutic dose to produce lifespan extension. These results led us to conclude that all drugs should be tested at concentrations of 1 and 10 µM.

**Fig. 1:**
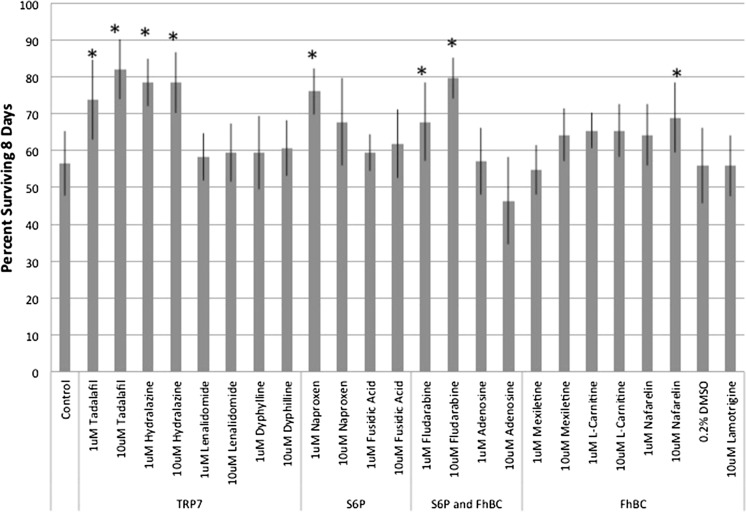
Percent rotifers surviving after 8 days exposure to drugs putatively binding to proteins of 3 rotifer temperature-sensing genes.* Asterisks* indicate treatments with significantly higher survival than controls (*P* < 0.05). (Color figure online)

As rotifers are aquatic animals, all exposures were with drugs dissolved in the medium. The effects of 12 drugs on the survival of *B. manjavacas* are shown in Fig. 1. Survival after 8 days of continuous drug exposure was compared to either the control (dilution medium) or a solvent control (0.2 % DMSO), if the drug required DMSO to dissolve in the test medium. Survival was similar in both the control and solvent control. Exposure to two drugs putatively binding to the TRP7 protein, tadalafil and hydralazine at 1 and 10 µM, significantly increased rotifer survival 31–43 % over the control. Similarly, 1 µM naproxen putatively bound to the S6P protein, significantly increasing survival 35 %. Fludarabine putatively binds to both the S6P and FhBC proteins and significantly increased rotifer survival by 21 and 43 % at 1 and 10 µM, respectively. Nafarelin at 10 µM putatively bound to the FhBC protein and significantly increased rotifer survival by 22 %.

**Fig. 2:**
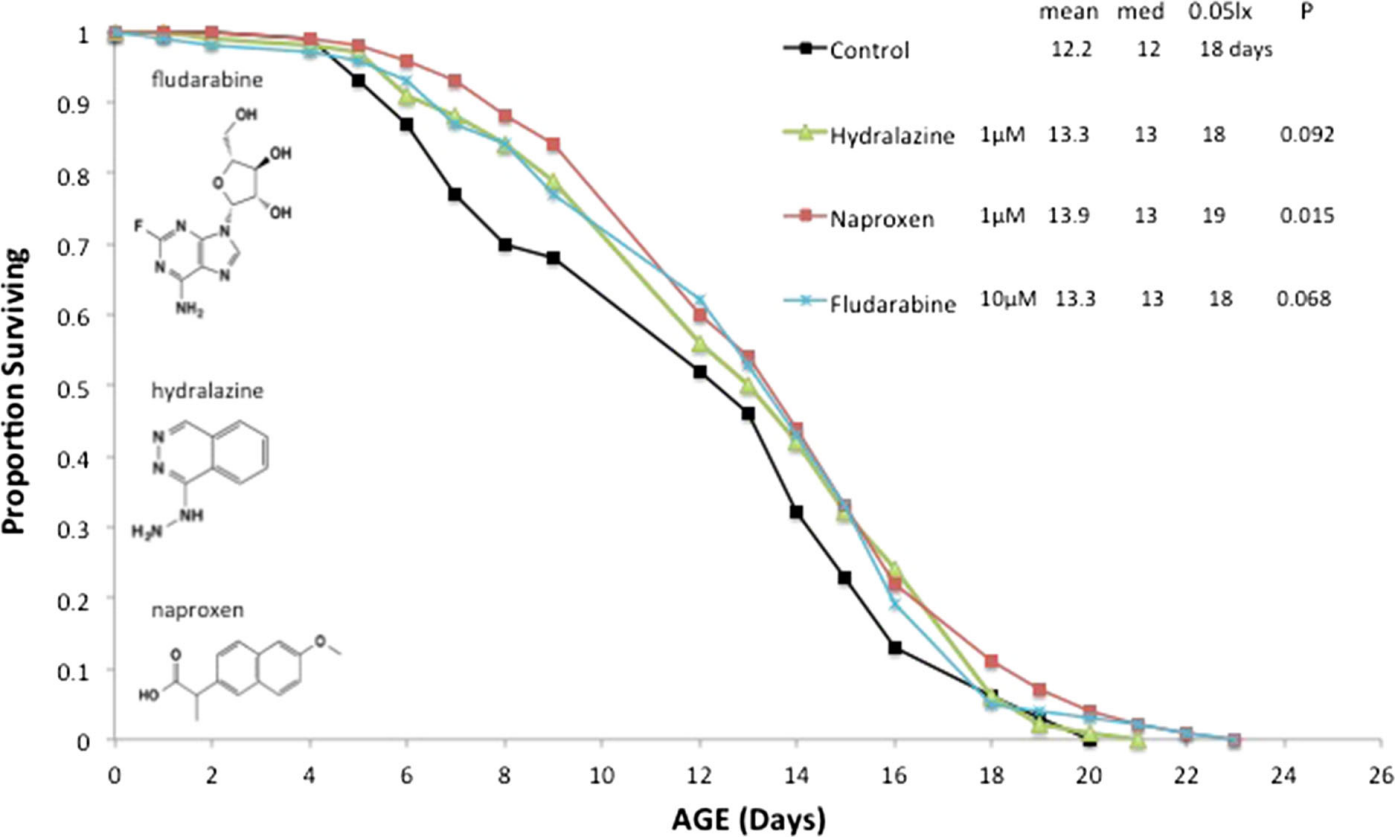
*B. manjavacas* survival in treatments of continuous 1 uM hydralazine, 1 µM naproxen or 10 µM fludarabine exposure. Proportion surviving refers to the age-specific survival from an initial cohort of 120 rotifers. Mean, median and age in days when 5% of the cohort remains alive is shown in the figure legend.* P* refers to the probability that the survival curve differs significantly from the control by Wilcoxon’s test. (Color figure online)

**Fig. 3:**
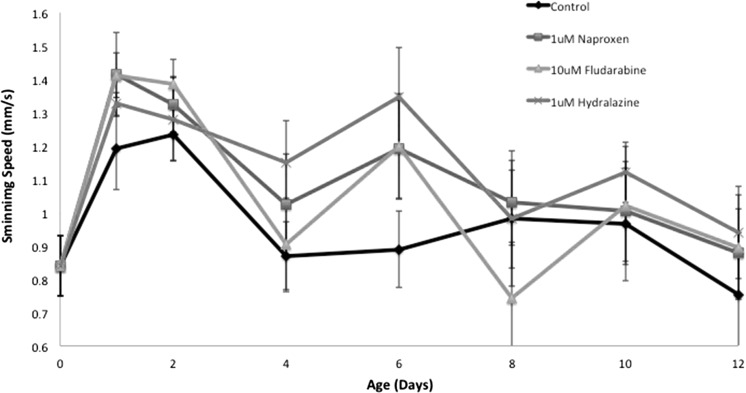
Effect of continuous exposure to 1 µM naproxen, 10 µM fludarabine, or 1 µM hydralazine on delaying the age-induced decline of rotifer swimming speed. (Color figure online)

**Fig. 4:**
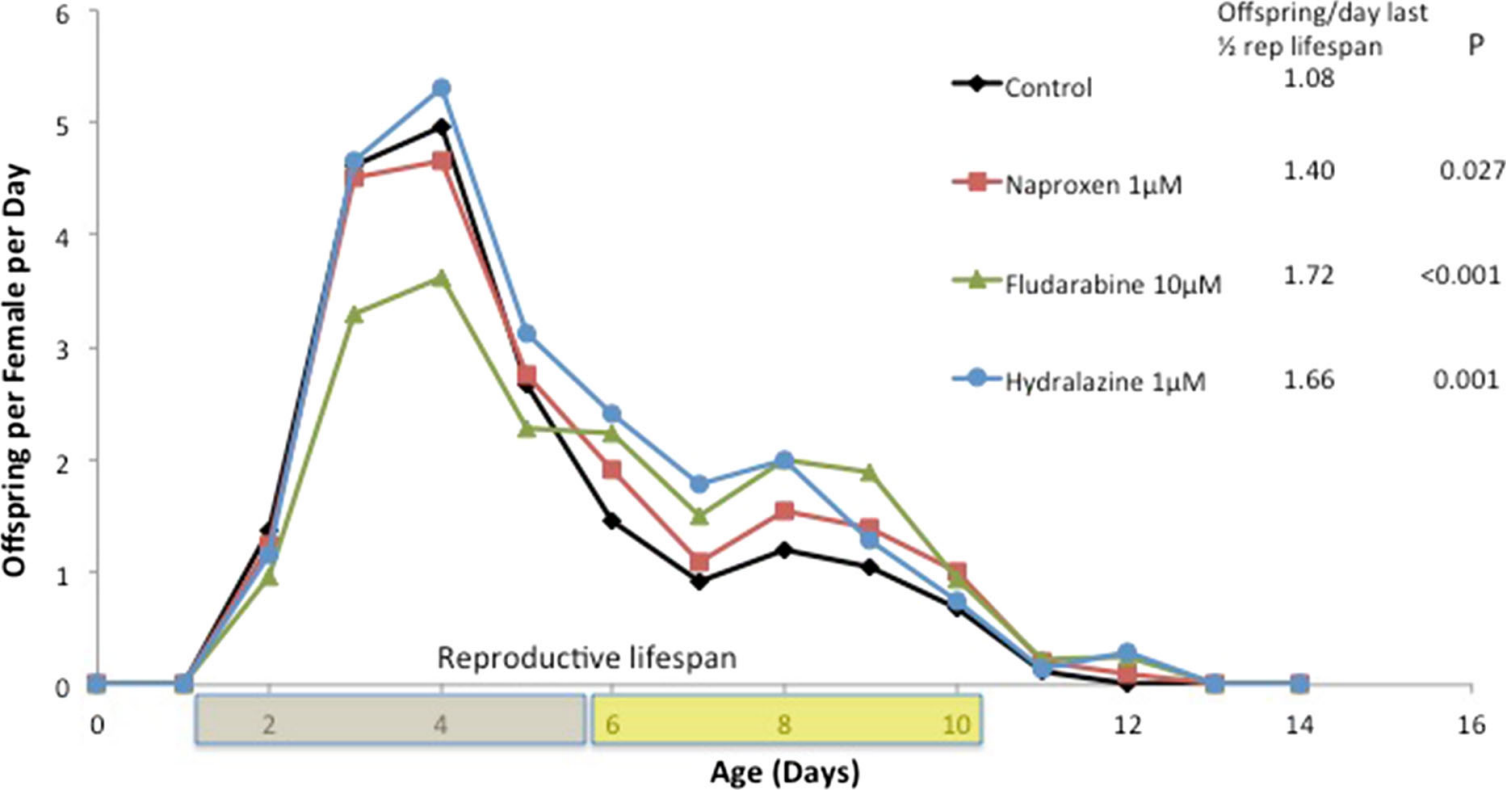
Effect of drugs on age-specific asexual reproduction of* B. manjavacas* females. Reproductive lifespan is divided into the first half (gray bar) and second half (yellow bar). Offspring produced per day of the second half of the reproductive lifespan is listed in the figure legend.* P* refers to the probability that offspring production is significantly higher than control by ANOVA and Dunnet’s test. (Color figure online)

**Fig. 5:**
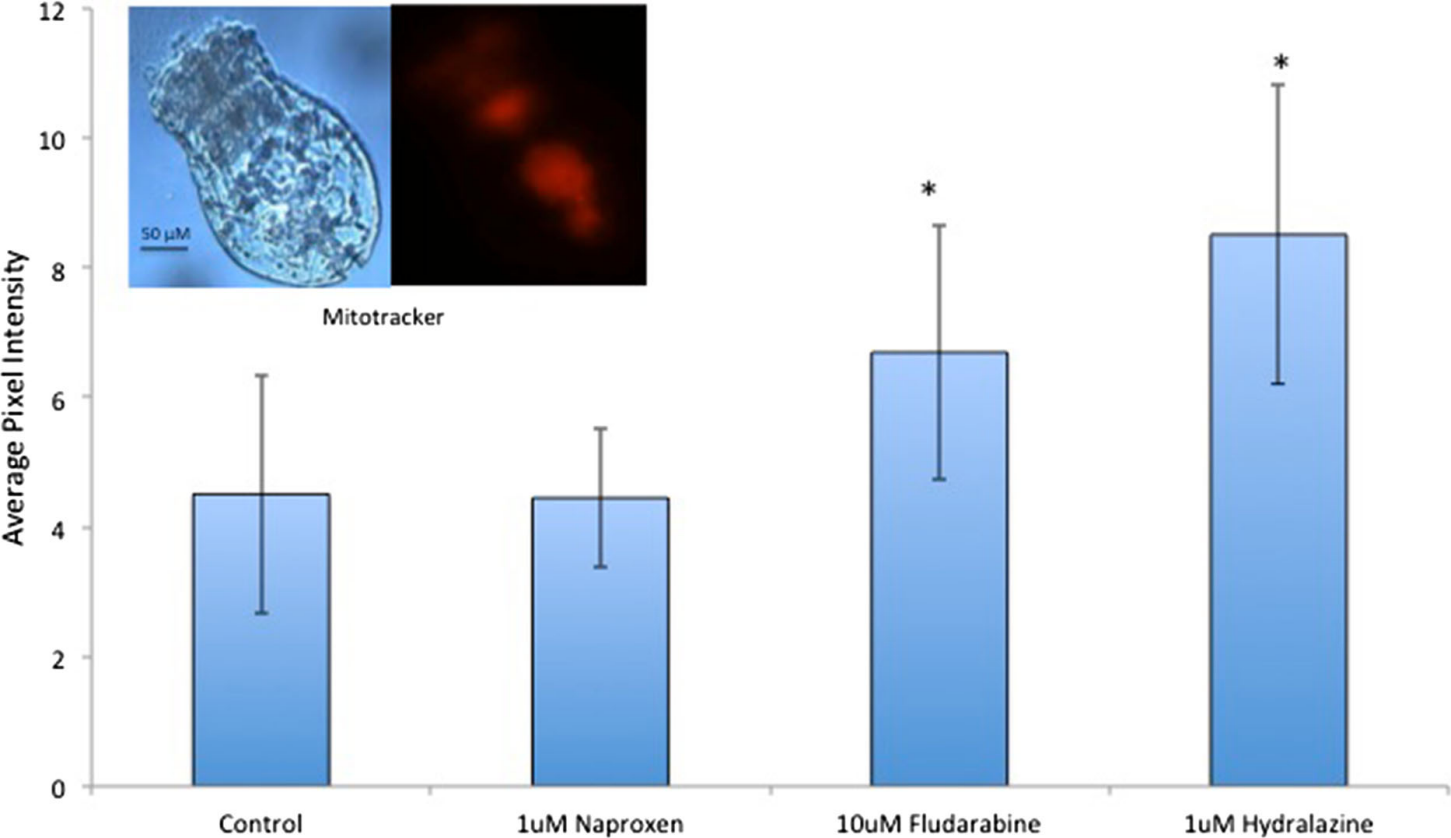
Fludarabine and hydralazine enhance mitochondrial activity at age 4 days.* Y*-axis is average pixel intensity of fluorescence at 620 nm.* Asterisks* indicate the probability by ANOVA and Dunnet’s test that the treatments have higher fluorescence than the control. (Color figure online)

**Fig. 6:**
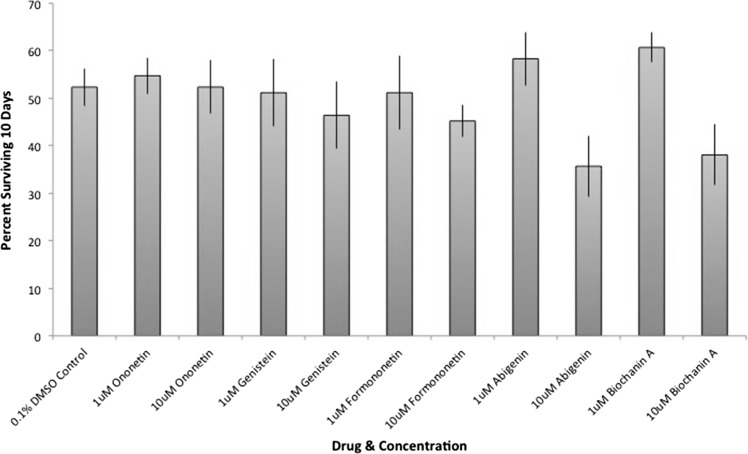
10-day survival screen for 5 ononetin analogs. (Color figure online)

The results from the 8 day survival screen were followed up in a full cohort life table experiment (Fig. 2). Cohorts of 120 females were continuously exposed to 1 µM hydralazine, 1 µM naproxen, or 10 µM fludarabine, and compared to survival in a no drug control. The naproxen treatment significantly extended rotifer mean lifespan by 14 %, whereas the lifespans of hydralazine and fludarabine exposed rotifers were not significantly different from controls. One explanation for differences in the results of the 8 day survival screen and the life table experiment, is that the former was conducted at 28 °C, whereas the latter was conducted at 22 °C.

In addition to life extension, we also are interested in drugs capable of extending rotifer healthspan. We have developed three endpoints that estimate rotifer health: swimming speed (mobility proxy), reproduction (overall vitality), and mitochondria activity (cellular senescence proxy). *B. manjavacas* females swim continuously throughout their life, initially at an average of 0.84 mm/s as juveniles, increasing to 1.23 mm/s at age 2 days, followed by a decline back to 0.86 mm by age 4 days (Fig. 3). Swimming speed remains steady at about 0.9 mm/s through age 10 days, and then slowly declines until senescent females stop swimming, fall to the bottom and die at age about 14 days. When treated with naproxen, fludarabine or hydralazine, maximum swimming speed peaked at age 1.5 days at 1.38, 1.38 and 1.28 mm/s, respectively. The decline in swimming speed was more gradual in the drug treatments, so that on day 6, mean swimming speed of females was 1.19 mm/s for naproxen, 1.20 for fludarabine, 1.35 for hydralazine, as compared to 0.88 mm/s in the control. These differences are significant by an F-test, with P values of 0.038–0.040. These data suggest that treatment with these three drugs slows the decline in rotifer swimming speed in older age classes.

Reproduction is an endpoint that often correlates with female health and vitality. In toxicology tests, rotifer reproduction typically is suppressed by toxicant stress at lower concentrations than effects on survival are observed (Snell and Janssen 1995). After a brief juvenile period of 1 day, rotifer females begin asexual reproduction and continue until age 12 days, so that their reproductive lifespan is about 80 % of their entire lifespan (Fig. 4). Reproduction peaks on day 4 at about five offspring/female/day and then gradually declines until reproduction ceases on day 12. Their reproductive lifespan can be divided into two halves, days 1–5 and days 6–10. We compared the effects of the drugs naproxen, fludarabine and hydralazine on reproduction in the second half of the reproductive lifespan. The rationale is that if these drugs improve the health of females in older age classes, we might observe higher reproductive rates. The average number of offspring produced by control females in the second half of their reproductive lifespan was 1.08 per day. In contrast, females treated with 1 µM naproxen produced 1.4 offspring per day. Likewise, females treated with 10 µM fludarabine or 1 µM hydralazine produced 1.72 and 1.66 offspring per day, respectively. All three of these reproductive rates are significantly higher than control with P values of 0.027, <0.001, and 0.001, respectively.

 The third endpoint that we used to estimate rotifer healthspan is mitochondria activity. Using the fluorochrome Mitotracker as a proxy for overall mitochondrial activity, we quantified the decline in fluorescence in older age classes. Preliminary experiments indicated that declines in mitotacker fluorescence can be seen at age 4 days, about 1/3 of the rotifer lifespan. *B. manjavacas* treated with 1 µM hydralazine or 10 µM fludarabine retained 49 % (P = 0.038) and 89 % (P = 0.002) greater mitochondria activity, respectively, than untreated controls (Fig. 5).

## Discussion

 The significance of these results is that they provide a proof of concept for coupling computation to experimentation to quickly identify new drug candidates with anti-aging potential. Exploring the pool of FDA approved drugs significantly shortens drug development cycles because the safety these compounds in humans is already established (Ashburn and Thor 2004). Drug repurposing also reduces the cost and associated risks typical of traditional drug discovery (Pantziarka et al. 2014). Most drugs bind to multiple targets (Zhou et al. 2015), opening possibilities that they have as yet undiscovered effects beyond their licensed targets. A sensible place to identify new targets for novel anti-aging therapies is in the physiological processes that underlie aging. Even if lifespan extension is not observed, certain drugs could improve healthspan, slowing age-dependent losses in mobility and vitality. Screening drugs for anti-aging effects using a rotifer bioassay is a powerful first step in identifying compounds worthy of follow-up in vertebrate models (Fig. 6).

The feasibility of structure-based drug repurposing has been demonstrated by Taylor et al. (2014). They identified druggable targets for the platelet collagen receptor, GPVI, which promotes pathological thrombus formation through inappropriate platelet aggregation. If small molecules could be found that modulate platelet formation with minimal side effects, the impact of cardiovascular disease may be mitigated. To find drugs that bind to GPVI, they used a computer model that docked a FDA-approved drug library into the GPVI collagen-binding site. They found that the drugs losartan and cinanserin inhibited GPVI-mediated platelet activation selectively, competitively, and dose-dependently. Even though losartan is licensed as an angiotensin II receptor antagonist drug used to treat high blood pressure, it also binds to GPVI receptor. Similarly, cinanserin is a 5-HT_2A_ and 5-HT_2C_ receptor antagonist, used to treat atypical pneumonia, but also binds to GPVI. The efficacy of these two drugs as antiplatelet agents needs further testing, but their potential for this new therapeutic use was rapidly identified by computer modeling followed by experimental testing on isolated human platelets.

Since many aging pathways have yet to be identified, it is sensible to screen a wide range of drugs for anti-aging effects. A variety of drug screens have been conducted using *Caenorhabditis elegans* (Collins et al. 2006), with varying degrees of success. Evason et al. (2005) screened 19 FDA-approved drugs and identified ethosuximide, an anticonvulsant in humans, which extended mean lifespan in wild-type *C. elegans* by 17 %. Ethosuximide and trimethadione exposure at 4 mg/mL also delayed functional declines in body movement and pharyngeal pumping. Kumar et al. (2016) screened 15 FDA-approved drugs for their effects on the lifespan of *C. elegans* and found that exposure to 2.4 mm of the antihypertensive drug captopril extended lifespan 23 %. In both of these studies, drugs with mechanisms of action thought to impact aging pathways were chosen for screening. Since many aging pathways remain unknown, this approach is unavoidably biased toward pathways already characterized. Kwok et al. (2006) described a method to conduct a screen of 14,100 small molecules for bioactivity and found that 308 produced morphologically recognizable phenotypes in *C. elegans*. Although this was an unbiased high-throughput screen, none of these phenotypes could be related to aging processes. The strength of using FINDSITE^comb^ to prioritize drugs for screening for anti-aging effects as we did in this study is that this algorithm provides a ranked list of small molecules to target proteins. If one screens the top 50 molecules, roughly 21 % will have binding affinities in the micromolar range or better (Srinivasan et al. 2014). Choosing drugs to screen based on their binding properties is more likely to produce novel hits than screening drugs based on their putative modes of action in known aging pathways.

 Work by Johnston and Snell (2016) implicated three novel rotifer genes in the determination of lifespan and healthspan. These observations motivated us to screen a library of FDA drugs for compounds that are predicted to bind to proteins produced by these genes. Our intent was to determine whether some of these drugs could be repurposed for anti-aging therapy. Predicted binding partners were experimentally assessed for anti-aging efficacy to identify new drug targets. For example, the transient receptor potential cation channel gene family (TRP) is a non-selective calcium permeant cation channel involved in osmotic, mechano- and thermosensitivity (Virens et al. 2004). It regulates intracellular Ca^2+^ levels, maintenance of functional intercellular barriers, expression of chemokines and cytokines related to proinflammatory pathways in adipocytes, and production of IL-8 in mice (Kottgen et al. 2008). Although TRPs play several vital roles in regulating cell metabolism, they have not been implicated in aging. Likewise, ribosomal protein S6 kinase alpha-1 (S6 K) is a family of protein kinases involved with signal transduction and the regulation of several cellular pathways (Frödin and Gammeltoft 1999). In mice their putative function is to mediate the activation of several mitogenic and stress-induced transcription factors. These in turn mediate cell proliferation, survival, and differentiation by modulating mTOR signaling and repressing pro-apoptotic function of BAD and DAPK1 proteins. mTOR is a well known aging pathway, but the role of S6 K in its regulation is poorly understood. Proteins coded by forkhead box C genes are transcriptional activators that regulate a variety of developmental pathways in humans, including organ growth, artery morphogenesis and blood vessel remodeling (van der Horst and Burgering 2007). These proteins also affect insulin-signaling pathways, but again their connection to aging is not well understood. The significance of this work is that these three genes have been identified as potential targets for anti-aging drug therapy and several candidate drugs have been experimentally identified that are capable of extending lifespan or healthspan in a rotifer animal model.

The FINDSITE^comb^ algorithm identified several binding partners for the TRP, S6 K and FhBC proteins and focused our attention on particular drugs. Naproxen is a nonsteroidal anti-inflammatory drug (NSAID) for relieving pain, fever, swelling, and stiffness. It is a nonselective COX inhibitor, reversibly inhibiting both the COX-1 and COX-2 enzymes (Hinz et al. 2008). Naproxen also may have anti-viral activity against influenza by blocking the RNA-binding groove of viral nucleoprotein, thus preventing the formation of ribonucleoprotein complexes (Lejal et al. 2013). Although we do not know whether these same mechanisms of action apply to rotifers, continuous treatment with 1 µM naproxen extended rotifer lifespan by 14 %. Fludarabine is a chemotherapy drug used to treat hematological malignancies like chronic lymphocytic leukemia (Rai et al. 2000). It is a purine analog, that inhibits DNA synthesis by interfering with ribonucleotide reductase and DNA polymerase. Exposure of rotifers to 10 µM fludarabine significantly improved reproduction and mitochondrial function in older age classes, these two endpoints serving as overall vitality and cellular senescence proxies, respectively. Hydralazine is an antihypertensive drug that relaxes smooth muscle, causing vasodilation in arteries and arterioles (Candelaria et al. 2010). This promotes a decrease in peripheral resistance and lowers blood pressure. It also has therapeutic value as a DNA methyltransferase inhibitor. Treatment of rotifers with 1 µM hydralazine slowed the loss of mobility (swimming speed) with aging. There has been no prior indication that any of these three drugs might be useful for anti-aging therapy. Our work identified these three drugs as potentially useful for anti-aging therapy from thousands of FDA approved compounds. Follow up studies of the efficacy of these drugs in slowing aging in vertebrate models is clearly warranted. But taken as a whole, these studies demonstrate the utility of FINDSITE^comb^ as a prioritization tool to identify repurposed FDA approved drugs (as well as new chemical entities) that might show promising anti-aging properties. When combined with a screen in a model animal such as rotifers promising leads can be rapidly identified which can then be subject to subsequent testing in vertebrate animal models.
